# MYC: there is more to it than cancer

**DOI:** 10.3389/fcell.2024.1342872

**Published:** 2024-03-06

**Authors:** Mariano F. Zacarías-Fluck, Laura Soucek, Jonathan R. Whitfield

**Affiliations:** ^1^ Models of Cancer Therapies Laboratory, Vall d’Hebron Institute of Oncology (VHIO), Vall d’Hebron Barcelona Hospital Campus, Barcelona, Spain; ^2^ Department of Biochemistry and Molecular Biology, Universitat Autònoma de Barcelona, Bellaterra, Spain; ^3^ Institució Catalana de Recerca i Estudis Avançats (ICREA), Barcelona, Spain; ^4^ Peptomyc S.L., Barcelona, Spain

**Keywords:** MYC, targeting, therapy, non-oncological diseases, transcription factor

## Abstract

MYC is a pleiotropic transcription factor involved in multiple cellular processes. While its mechanism of action and targets are not completely elucidated, it has a fundamental role in cellular proliferation, differentiation, metabolism, ribogenesis, and bone and vascular development. Over 4 decades of research and some 10,000 publications linking it to tumorigenesis (by searching PubMed for “MYC oncogene”) have led to MYC becoming a most-wanted target for the treatment of cancer, where many of MYC’s physiological functions become co-opted for tumour initiation and maintenance. In this context, an abundance of reviews describes strategies for potentially targeting MYC in the oncology field. However, its multiple roles in different aspects of cellular biology suggest that it may also play a role in many additional diseases, and other publications are indeed linking MYC to pathologies beyond cancer. Here, we review these physiological functions and the current literature linking MYC to non-oncological diseases. The intense efforts towards developing MYC inhibitors as a cancer therapy will potentially have huge implications for the treatment of other diseases. In addition, with a complementary approach, we discuss some diseases and conditions where MYC appears to play a protective role and hence its increased expression or activation could be therapeutic.

## 1 Discovery and initial characterisation of MYC

The *c*-*MYC* gene encodes for a basic helix-loop-helix protein that acts as a pleiotropic transcription factor. It was discovered more than 40 years ago by the pioneering work to isolate and characterise avian retrovirus MC29, which showed its oncogenic potential, followed by the discovery of *c-MYC,* its cellular homolog identified from the chicken genome (Duesberg et al., 1977; Sheiness et al., 1978; Hu et al., 1979; Abrams et al., 1982; Vennstrom et al., 1982; Hann et al., 1983; Dang et al., 1989). Later studies discovered two human paralogs with overlapping roles and a more limited tissular expression: MYCN, or N-MYC, identified in Neuroblastoma cells, and MYCL, or L-MYC, found in Lung carcinoma cells, respectively, reviewed in (Massó-Vallés et al., 2020). c-MYC (from now on, MYC) and its paralogs share an N-terminal transactivation domain (TAD), capable of interacting with a plethora of proteins regulating chromatin remodelling, transcription and MYC stability, a central region, and a C-terminus basic helix-loop-helix (bHLH) domain (Beaulieu et al., 2020). The latter initially pointed to MYC as a protein capable of binding DNA, although it was not until 1990 that it was discovered that MYC bound the sequence CACGTG (termed the E-box) (Prendergast and Ziff, 1991). Shortly after, a MYC dimerisation partner was identified: MYC-associated protein X, MAX a bHLH-Zip protein, specifically associated with c-MYC and its paralogs. Using a yeast model, DNA binding and transcriptional transactivation by MYC were found to be both dependent on this heterodimer (Blackwood and Eisenman, 1991; Amati et al., 1992), and a study in *Drosophila*, revealed that dMyc, dMax and the Max-binding protein dMNT could bind up to ∼15% of the coding regions in the fly genome (Orian et al., 2003).

## 2 Physiological processes mediated by MYC

**FIGURE 1:**
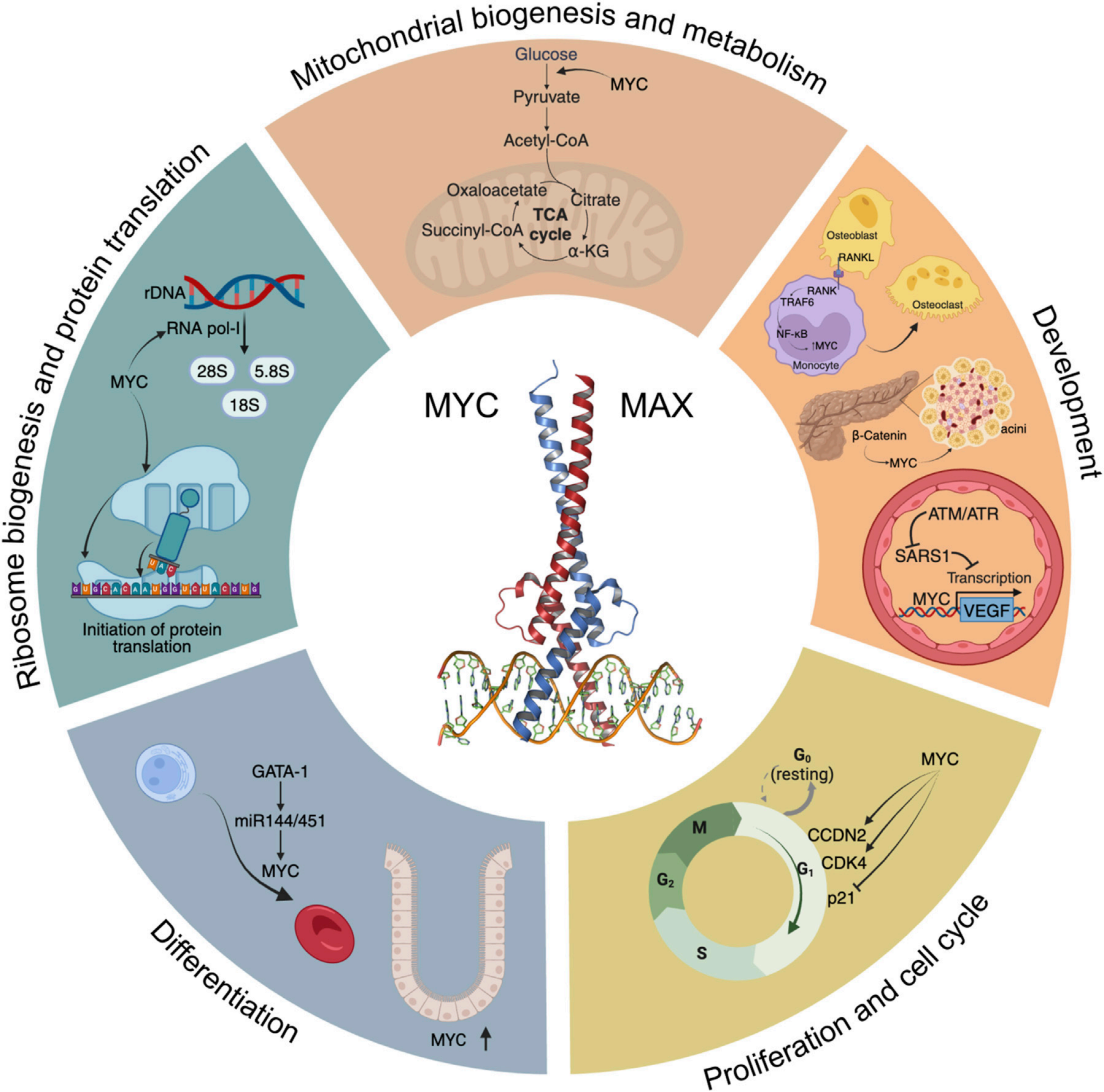
A “Hallmarks” style representation of MYC’s physiological functions. Some of the main signalling pathways and processes modulated by MYC are depicted, including glycolysis and mitochondrial biogenesis, development, cell cycle progression, differentiation, ribosome biogenesis and initiation of protein translation.

In this section we describe how MYC plays a key role in multiple aspects of the biology of cells and tissues. This is also summarised in Figure 1.

### 2.1 Proliferation

MYC’s most established role under physiological conditions is to promote efficient proliferation (Jha et al., 2023). This has been studied in many model systems and organisms from mammalian tissues to flies. Although MYC is virtually undetectable in quiescent cells, upon mitogenic or serum stimulation, MYC levels are induced, and cells enter the G1 phase of the cell cycle through MYC-dependent upregulation and/or activation of key mediators of cell cycle progression, such as CCDN2, CDK4, and the CyclinE2-CDK2 complex, degradation of p27 (Kip1, encoded by CDKN1B), and repression of p21 and p15 (encoded by *CDKN1A* and *CDKN2B*, respectively) among others (Pelengaris and Khan, 2003). In contrast, MYC-dependent repression was described to be mediated by MYC-MAX interaction with Miz-1 (Staller et al., 2001). Expression of MYC is necessary, and in some cases sufficient, for inducing cell proliferation. In fact, ectopic expression of MYC locks cells in a continuously proliferating state, even in the absence of mitogens (Evan et al., 1994). This is probably the evolutionary basis for the tight regulation of MYC expression, which is in stark contrast to the ubiquitous expression of its binding partner MAX.

MYC’s key role in proliferation and growth is highly conserved throughout the animal kingdom, with a presence in invertebrates such as *Drosophila,* where dMYC is the only paralog, whose loss impacts on cellular growth and size. Its overexpression promotes G1/S progression but not cell division, which is dependent on other players (Johnston et al., 1999). Interestingly, expression of dMYC is able to rescue the proliferation defects in mouse embryonic fibroblasts deficient for MYC, although it does not affect cell growth. Thus, MYC and dMYC have similar biological functions, but their outcomes depend on specific cell targets (Trumpp et al., 2001). Given this ancestral conservation, it is curious that MYC itself was lost during the evolution of *C.elegans,* which, instead, retains orthologous MAX and MLX networks (Yuan et al., 1998; Gallant, 2006; McFerrin and Atchley, 2011).

### 2.2 Differentiation

A key role for MYC in differentiation has been demonstrated in many tissues. One prominent example is found in the hematopoietic system, where MYC is involved in the expansion of committed progenitors by controlling the balance between self-renewal and differentiation through the modulation of stem cell migration and/or adhesion to the niche. MYC was described as necessary to induce the first differentiation steps in these murine stem cells, whereas in committed progenitors, MYC is required for proliferation and expansion (McFerrin and Atchley, 2011). Additionally, gene expression analyses using Krüppel-like factor 1 (KLF1), a master regulator of adult erythropoiesis (Perkins et al., 2016), and KLF2 knockout mice identified MYC as a central node in a network of genes controlled by both KLF1 and KLF2. Ablation of MYC in primitive proerythroblasts showed that its absence resulted in a block in the normal expansion of erythroid cells (Pang et al., 2012). In addition, the master regulator of haematopoiesis GATA-1 represses MYC transcriptional activity through binding to MYC’s promoter or through activation of miR-144/451, inducing proliferative arrest, thus facilitating erythroid differentiation (Rylski et al., 2003). Conversely, depletion of miR-144/451 blocks erythroid differentiation through de-repression of MYC (Xu et al., 2020). This GATA-1-miR144/451-MYC axis controls normal erythroid differentiation.

Another example of MYC’s role in differentiation can be found in murine embryonic stem cells (mESC), where MYC inhibits the expression of differentiation-specific genes through modulation of a set of miRNAs that attenuate their proliferation (Lin et al., 2009). Its inhibition or deletion strongly curbs transcription, splicing and protein synthesis, leading to a proliferative arrest, reminiscent of embryonic diapause. Remarkably, this arrest is reversible and does not compromise cell pluripotency (Scognamiglio et al., 2016). Additionally, MYC maintains the pluripotent transcriptome by amplifying the transcription of a large set of genes during the transition from mESC to the totipotent two-cell-like state (Fu et al., 2019), and is also important in metabolic and epigenetic regulation of mESCs during mouse embryonic development (Fan and Li, 2023).

In human adipose tissue, MYC was identified as a significant regulator of adipose stem cell differentiation, which is necessary for the maintenance and function of the tissue. MYC is induced by glucocorticoid in the early stages of differentiation and precedes the downregulation of key suppressor genes as well as the induction of functional effectors (Deisenroth et al., 2014).

In crypt development in the small intestine in the mouse, MYC signalling pathways are significantly enriched. Laser capture microdissection followed by functional genomics analysis of epithelial progenitors showed an enrichment, with respect to normal crypt base epithelium, of a series of transcripts encoding for proteins that regulate MYC transcription, protein stability, and transactivation of its target genes (Stappenbeck et al., 2003). Subsequent studies, however, showed that MYC is necessary for normal crypt formation, but does not affect cell proliferation or fate of already formed crypts (Bettess et al., 2005).

A role in differentiation is present in *Drosophila* too, where dMYC is required for intestinal stem cell maintenance, proliferation, and lineage differentiation during tissue homeostasis (Ren et al., 2013). Also in *Drosophila*, IGF2BP stabilises *MYC* mRNA, increasing its protein levels, leading to larger neural stem cells and faster division rates (Samuels et al., 2020). In line with this, GSK3-α and -β differently regulate cortical development through MYC (Ma et al., 2017), while MYC inhibits the differentiation of neural progenitor cells into neurons (Wang et al., 2020).

### 2.3 Ribosome biogenesis and protein translation

Ribosome biogenesis involves the synthesis and processing of ribosomal RNA (rRNA) proteins, the assembly of ribosomal subunits and their export to the cytoplasm, and it requires the coordinated activities of the three nuclear RNA polymerases (RNA pol I, II and III). Not surprisingly, MYC regulates multiple stages of ribosomal biogenesis through RNA pol I-mediated transcription of rRNA, RNA pol II-dependent transcription of ribosomal protein genes and translation initiation factors, among others (reviewed in van Riggelen et al., 2010). Using Crispr-Cas9-based reverse genetics to dissect the transcriptional networks downstream of MYC *in vivo*, it was shown that MYC’s ability to drive growth depends on its ability to upregulate ribosome biogenesis (Zielke et al., 2022). Consistent with this, inducible overexpression of MYC stimulates both ribosome biogenesis and protein synthesis (Mori et al., 2021).

Intimately related to ribosome biogenesis, protein translation is a critical process on which cell growth and division depend. It is regulated at different levels, although the key point of control seems to be the initiation of translation, which involves the translation initiation factor eIF4E binding to the 7-methyl guanosine cap at the 5′ end of mRNAs (Schmidt, 2004). Experiments carried out with *MYC* knockout rat fibroblasts showed that levels of protein translation, a mechanism that is under control of mammalian TOR complex 1 (mTORC1) (Ma and Blenis, 2009), are two-to-three fold higher in MYC wild-type when compared to MYC^−/−^ cells (Mateyak et al., 1997). Microarray analysis of these cells showed that the largest category of MYC-induced genes was involved in protein translation, where an impressive 60% of the genes were upregulated by MYC (Guo et al., 2000). Additionally, a specific role in regulating eIF4E was confirmed after showing its expression correlated with and was regulated by MYC (Rosenwald et al., 1993). Indeed, MYC binds to two canonical E-boxes in the *eIF4E* promoter and is necessary for its expression. Importantly, inhibition of eIF4E was able to block MYC-induced transformation (Lynch et al., 2004).

The link between MYC and ribosomes is conserved in flies and even in the multicellular eukaryote *Nemostella* (Brown et al., 2008; Stine et al., 2015). Indeed, in *Drosophila,* expression of dMYC is necessary and sufficient to control rRNA synthesis and ribosome biogenesis (Grewal et al., 2005) and physiological dMYC targets, whose promoters are enriched in the E-box motif (frequently in the first 100 nucleotides following the transcription start site), play a role in nucleolar function and ribosome biogenesis (Hulf et al., 2005).

### 2.4 Metabolism and mitochondrial biogenesis

The first evidence of the *in vivo* induction of glycolysis by MYC was provided using transgenic mouse models where MYC was overexpressed in hepatocytes under the control of phosphoenolpyruvate carboxykinase. Transcriptional analyses of livers from these transgenic mice revealed increased expression of the glycolytic enzymes of glucokinase, PFKFB1, pyruvate-kinase L, and the glucose transporter GLUT2, which resulted in increased glycolysis compared to controls (Hulf et al., 2005). Later on, MYC was shown to induce a collection of glycolytic genes including ALDOA, ENO1, GAPDH, GPI, LDHA, HK2, PFKM, PGK1, PKM, and TPI1 (Kim et al., 2004), confirming the key role of MYC in controlling metabolism.

Importantly, MYC’s effect on metabolism becomes more evident when MYC is absent. Even in the presence of adequate energy-generating substrates, *MYC*-knockout fibroblasts remain ATP-depleted and respond by activating AMPK, in an attempt to remedy this energy deficit. However, since AMPK activation leads to upregulation of glycolysis and oxidative phosphorylation, both dependent on MYC, the AMPK response fails and the cells, unable to correct the energy production, remain slowly proliferating (Edmunds et al., 2014).

A final well-established role for MYC is in the mitochondria biogenesis. Using a combination of *in vitro* and *in vivo* MYC-modulating models, a role was shown for MYC in regulating the expression of genes involved in mitochondrial structure, function, and biogenesis. These include TFAM, a key mitochondrial transcriptional factor and mtDNA replication factor. These results point to MYC’s role as a master mitochondrial switch coupling metabolic needs to cell growth and proliferation (Li et al., 2005). In this line, further work suggested that mitochondrial structure, function, and subcellular localisation are regulated over time, responding more rapidly to inactivation of MYC than to its activation. The increased mitochondrial mass induced by MYC was associated with increased organelle turnover, involving both fission and fusion proteins (Graves et al., 2012). Overall, these results reinforce the notion that MYC links cellular energy generation and proliferative needs.

MYC’s role in mitochondrial biogenesis is also conserved in *Drosophila*, where in the ovary, it stimulates gene expression, including that of many electron transport chain genes required for mtDNA replication and expression (Wang et al., 2019).

### 2.5 Development

An increasing number of studies point to a role for MYC in the development of multiple tissues and organs, including pancreas, bone, and blood vessels. Given the difference in phenotypes of the tissues, it is perhaps not surprising that the principal targets of MYC in each case are different. In fact, development of tissues is a phenotypic outcome of the physiological processes that MYC helps to control, so that MYC’s regulation of proliferation, differentiation and metabolism results in different phenotypic outcomes depending on the cell-specific gene expression, tissue type and body location. This highlights a reason why the definition of a single critical list of MYC target genes across different tissue contexts has proven impossible.

#### 2.5.1 Pancreas

The expansion of pancreatic acinar cells, the main components of pancreatic parenchyma, is promoted by β-catenin signalling, of which MYC is one key effector (Murtaugh et al., 2005). MYC’s importance in pancreas is stressed by the evidence that pancreatic inactivation of both *MYC* alleles in a mouse model leads to death after birth. Already at a late embryonic stage, these mice show a severe pancreatic hypoplasia, with poorly branched pancreatic ducts, disruption of exocrine pancreas formation and severe reduction of acini, characterised by reduced MYC target CDK4 expression and proliferation (Nakhai et al., 2008). These results were confirmed in an independent study using a mouse model with a 60%–70% reduction in MYC expression, in which pancreata showed fewer proliferating progenitors at E12.5, leading to significantly reduced pancreatic weight in two-month-old mice. Both arborization of the exocrine tree and acinar development were impaired at birth, but partially recovered at 2 months. Overall, MYC inactivation impairs normal acinar development and maturation, leading to the formation of lipid vacuoles in acinar cells, acquiring an adipocyte phenotype with aging (Bonal et al., 2009; Zhang et al., 2010).

#### 2.5.2 Bone

Bone remodelling results from the balance between two tightly regulated phenomena: osteoclastogenesis and osteogenesis. The osteoclast is a bone-resorbing cell with an origin in the monocyte-macrophage lineage (Yavropoulou and Yovos, 2008). As expected, MYC is involved in bone remodelling and is regulated by different signalling pathways. RANKL, a key cytokine expressed by osteoblasts, mediates osteoclastogenesis through a TRAF6-dependent NF-κB activation. Upon RANKL binding to monocytes, this cascade results in the induction of MYC, which is necessary for osteoclast differentiation, since its inhibition by the MYC dominant negative In373-Myc almost completely inhibits osteoclastogenesis (Battaglino et al., 2002).

On the other hand, inhibition of FOXO1, whose expression decreases upon RANKL-induced osteoclastogenesis, promotes the expression of MYC, while 10058-F4, a small molecular inhibitor of MYC, abrogates the osteoclastogenic effect of FOXO1 inhibition in a dose-dependent manner (Tan et al., 2015). Additionally, RANKL-induced expression of osteoclastogenic marker genes is significantly reduced in MYC-deficient osteoclast progenitors *in vitro*, but rescued by ectopic expression of MYC (Bae et al., 2017).

Finally, RNA-seq analysis of *MYC* wild-type and deficient bone marrow cells revealed that MYC regulates mTORC1 signalling. mTORC1 is activated at the early phases after RANKL treatment and suppressed at later phases of osteoclastogenesis, and this biphasic regulation is dependent on MYC. While inhibition of mTORC1 by rapamycin prior to RANKL stimulation almost completely prevented osteoclast formation, osteoclasts showed enhanced resorbing activity when mTORC1 was inhibited three days post-RANKL. In parallel, unfolded protein response (UPR) genes were found downregulated by MYC deficiency. In line with this, the expression of GADD34, a factor that regulates UPR and negatively regulates mTOR signalling, is increased in wild-type cells upon RANKL stimulation but not in *MYC*-deficient cells, and its deficiency partially restores RANKL-induced mTORC1 inactivation and suppresses osteoclastogenesis. Taken together, these data suggest that mTORC1 is suppressed in osteoclasts through a MYC/GADD34 axis (Bae et al., 2022). This is in stark contrast to protein translation, where MYC and mTORC1 jointly contribute to its regulation.

#### 2.5.3 Vascular development

MYC’s role in vascular development was confirmed by modulation of MYC levels. In fact, *c-MYC* knockout mice are embryonic lethal and have under-developed vasculature, that can be partially rescued by transgenic VEGF expression (Baudino et al., 2002). On the other hand, overexpression of MYC is also embryonic lethal due to multiple haemorrhagic lesions and defects in the vasculature, with concomitant elevated VEGF levels (Kokai et al., 2009). Thus, MYC and VEGF levels must be precisely controlled during early development. One key control involves Seryl-TRNA synthetase 1 (SARS1), which competes directly with MYC to control VEGF expression levels, and thus enables proper vasculature development (Shi et al., 2014). In hypoxic conditions, SARS1 is phosphorylated by ATM/ATR, and this impairs its DNA binding capacity, allowing MYC to induce VEGF expression (Shi et al., 2020).

## 3 Physiological MYC functions hijacked by tumour cells

**FIGURE 2:**
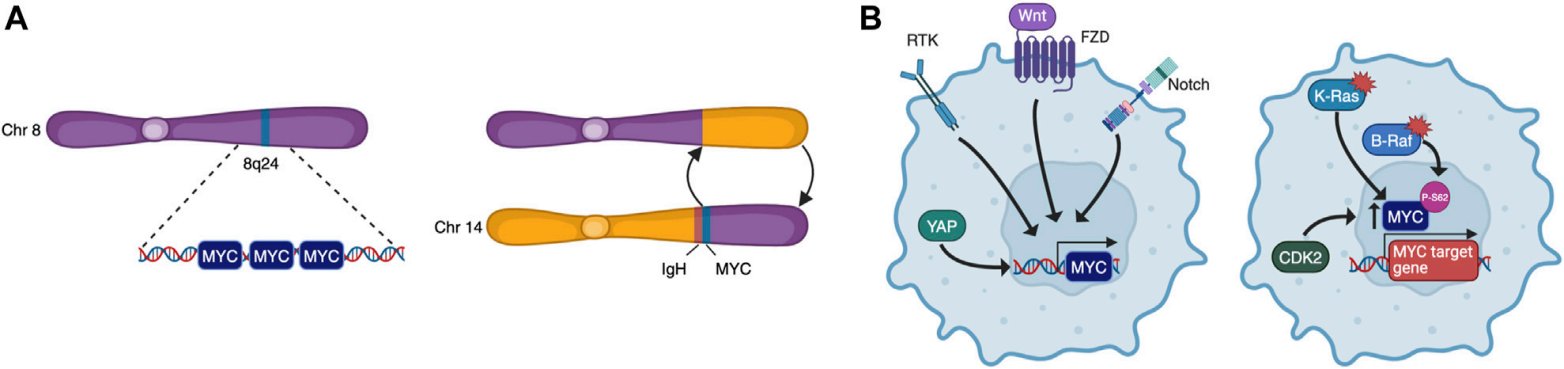
MYC is deregulated by multiple mechanisms. These include chromosomal rearrangements that lead to amplification or translocation **(A)**, and upstream signalling that causes increased transcription or protein stabilisation **(B)**.

To become fully transformed and tumorigenic, normal cells must overcome several barriers imposed on cell-autonomous programs such as cell cycle progression, DNA replication, evasion of senescence and apoptosis, as well as cell non-autonomous processes such as angiogenesis and immune surveillance. These constitute many of the Hallmarks of Cancer (Hanahan, 2022) and, as MYC may impinge on all these programs, it is a common target for oncogenic activation. Indeed, although its expression is tightly regulated in normal cells, cancer cells are almost unavoidably characterised by deregulated MYC activity. This can be the result of many different processes such as gene amplification, translocation (Figure 2A), epistasis, epigenetic changes, upstream signalling, and increased protein stability (Figure 2B) (Dhanasekaran et al., 2022). Oncogenic MYC promotes tumorigenesis in different yet complementary ways, co-opting many of the physiological processes described above. Its deregulation is associated to uncontrolled proliferation, rewiring of cellular metabolism, increased ribosomal and protein biogenesis and chromosome instability. MYC also affects cell non-autonomous hallmarks including reshaping of the tumour microenvironment, angiogenesis, induction of immunosuppressive cytokine release, and upregulation of immune checkpoint inhibitor proteins (Whitfield and Soucek, 2012; Dhanasekaran et al., 2022).

However, the sole overexpression of MYC is not sufficient for tumorigenesis in most cellular contexts. Indeed, MYC activation usually induces DNA replication and S phase entry without cellular division, hence cells become polyploid, accumulate DNA damage, and undergo proliferative arrest, senescence, or apoptosis depending on the cellular context (Gabay et al., 2014). This is why genetic alterations that circumvent the hurdles imposed by cell cycle checkpoints or apoptosis/senescence usually synergise with MYC overexpression to induce tumorigenesis. This was shown in seminal studies with transgenic mice harbouring tissue-specific inducible forms of MYC. For instance, in *MycER* ^*TAM*^ mice that express switchable MYC in pancreatic β-cells, MYC activation is sufficient to drive the cells into cell cycle, but unfettered proliferation is constrained by subsequent apoptosis, which quickly results in β-cell loss and diabetes. However, solely by co-expression of the anti-apoptotic protein Bcl-_XL_, MYC overexpression is then able to drive formation of pancreatic insulinomas (Pelengaris et al., 2002). Similarly, in adult mouse hepatocytes, conditionally expressed MYC leads to polyploidy in the absence of cell division, but concomitant reduction of p53 levels (by crossing with *TP53* ^*+/−*^ mice) resulted in increased tumorigenesis (Beer et al., 2004).

In this context, MYC expression levels seem an important determinant of the biological outcome. It was reported, for instance, that low levels of deregulated MYC can drive proliferation and oncogenesis by themselves, whereas apoptotic and p53 tumour suppressor pathways are only triggered above a certain MYC threshold (Murphy et al., 2008).

In summary, as observed in cancer but not limited to it, MYC’s role in promoting multiple physiological processes means that its overexpression, deregulation, or insufficiency can lead to an array of human diseases and disorders.

## 4 MYC’s involvement in diseases and conditions

### 4.1 Metabolic diseases

Metabolic dysfunction-Associated steatotic liver disease (MASLD), previously known as non-alcoholic fatty liver disease, is strongly associated with obesity and insulin resistance (Browning and Horton, 2004), as well as with increased mortality and cardiovascular disease burden (Kim et al., 2021). It begins with the aberrant accumulation of triglycerides in the liver (steatosis) and can proceed to a Metabolic Dysfunction-Associated Steatohepatitis (MASH), which in turn, can eventually give rise to cirrhosis and liver cancer.

*Alb-myc* ^*tg*^ mice overexpress MYC in hepatocytes, and at 36 weeks, they spontaneously develop metabolic syndrome, characterised by obesity, hypertriglyceridemia, hyper-cholesterolemia, glucose intolerance and insulin resistance. The mouse livers show abnormal accumulation of lipids that leads to compensatory increased ß-oxidation that, in turn, generates oxidative stress. This results in, on the one hand, CD45^+^, F4/80^+^ immune infiltration, and on the other, increased hepatocyte apoptosis and compensatory proliferation. Hence, hepatic overexpression of MYC affects metabolism and leads to the development of mild steatohepatitis/fibrosis that progress to liver tumours with long latency. Moreover, MYC overexpression provides a pro-fibrotic tissue environment characterised by moderate but chronic hepatocyte apoptosis, pre-activation of hepatic stellate cells (HSCs) and high basal collagen expression. These HSCs have a high potential to proliferate and to produce extracellular matrix, especially after a second hit. This link between MYC and hepatic fibrosis is reinforced by the fact that MYC mRNA expression was found to be upregulated in patients with liver cirrhosis (Nevzorova et al., 2013).

Alcohol-associated liver disease (ALD) includes a variety of hepatic conditions from steatosis to cirrhosis. In patients with advanced stages of ALD, MYC is strongly upregulated and correlates with the progression of liver fibrosis. In line with this, wild-type mice fed with a Lieber-DeCarli (EtOH) diet showed higher MYC expression at the initial stages of liver injury and MYC remained elevated during the early phase of ALD progression.

**FIGURE 3:**
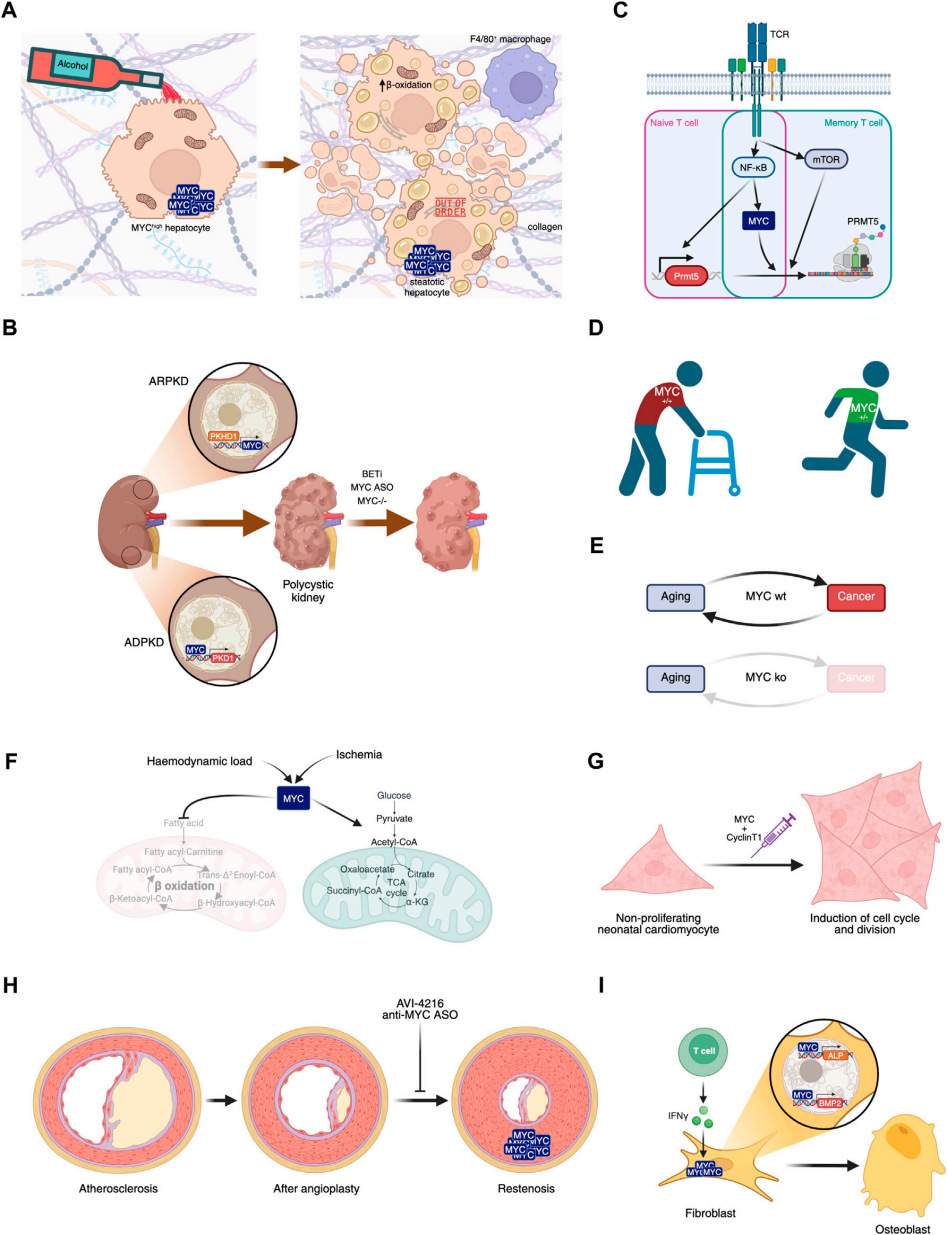
Involvement of MYC in diseases. **(A)** MYC overexpression in hepatocytes, in combination or not with alcohol consumption, leads to liver steatosis. **(B)** MYC’s role in polycystic kidney disease and its potential inhibition leading to disease amelioration. **(C)** MYC plays a central role in naive and memory T cell activation in Multiple Sclerosis. **(D)** MYC haploinsufficiency prevents aged-related phenotypes. **(E)** MYC knockout after weaning leads to aging in the absence of cancer, disrupting this biunivocal relationship. **(F)** Haemodynamic load or ischemia lead to MYC-dependent metabolic rewiring in the heart. **(G)** Transient expression of MYC and Cyclin T1 could have regenerative therapeutic impact in the heart after myocardial infarction. **(H)** Proliferation of smooth muscle cells leading to restenosis can be prevented by MYC inhibition. **(I)** IFN-γ-dependent MYC induction of ALP and BMP2 contribute to Ankylosing spondylitis symptoms.

MYC overexpression and alcohol consumption were further studied with *Alb-myc* ^*tg*^ mice. Following a 4-week Lieber-DeCarli diet, these mice presented deregulation of multiple disease-related pathways, and an increase in liver mass in the absence of proliferation, accompanied by hepatocyte hypertrophy, enhanced collagen deposition, increased mitochondrial oxygen radicals, and hepatic lipotoxicity. Mitochondrial and ER dysfunction caused metabolic effects involving glucose intolerance. Overall, MYC overexpression in the context of alcohol consumption led to impaired Akt-MDM2-p53 signalling that eventually may trigger ALD progression to fibrosis (Figure 3A) (Nevzorova et al., 2016). To our knowledge, studies to block MYC have not yet been performed in these models.

Intestinal MYC is also increased in humans and mice with obesity, likely due to activation of the ß-catenin pathway, of which MYC is a downstream target. In this case, its inhibition has been tested: intestinal-specific MYC disruption protected mice subjected to a high-fat diet against obesity, insulin resistance, hepatic steatosis and fibrosis (Luo et al., 2021). Overall, MYC plays multiple roles in obesity, including the maturation of progenitor cells, fatty acid metabolism and extracellular matrix remodelling. Of note, MYC modulates the inflammatory response, induces insulin resistance, and regulates intestinal dysbiosis (Nevzorova and Cubero, 2023).

Additionally, gerbils fed with a high-fat and high-cholesterol diet showed increased hepatic USP33 expression, whose modulation revealed a signal transduction pathway regulated by both this enzyme and MYC, which controls activation of HSCs, the main cells responsible for liver fibrosis (Ke et al., 2023). In this context, a potential drug treatment of MASH was recently identified: AZD3355, a GABA-B receptor agonist, proved to be anti-fibrotic, anti-inflammatory and hepatoprotective, and interestingly, MYC was identified as the top transcription factor regulated in HSCs treated *in vitro* with AZD3355 (Bhattacharya et al., 2021). All these data are all in line with a role for MYC in HSC activation and prompt the testing of MYC inhibitors in disease models.

Intriguingly, though, MYC expression in endothelial cells was shown to have a protective effect against diet-induced liver inflammation and fibrosis. *In vitro*, knockdown of MYC in human umbilical vein endothelial cells (HUVECs) induces cellular senescence accompanied by a proinflammatory senescence associated secretory phenotype (SASP) (Florea et al., 2013)^.^ *In vivo*, loss of endothelial MYC induced a significant increase in proinflammatory molecules CCL7 and osteopontin. Moreover, under a high fat diet, mice with *MYC* ^*−/−*^ endothelial cells showed transcriptional induction of inflammation-associated pathways characterised by an increase in neutrophil and macrophage infiltration and the secretion of chemo- and cytokines CCL11, CXCL1 and IL-17, all of which have a role in liver inflammation and MASH. Moreover, transcriptional analysis of endothelial cells from MYC knockout mice showed functions associated with liver hyperplasia/hyperproliferation and hepatocellular carcinoma. These findings are in line with scRNA analyses showing that endothelial *MYC* expression was downregulated in male cirrhotic livers compared to those of healthy individuals (Qi et al., 2022). Whether endothelial MYC knockdown *in vivo* induces senescence, which is related to inflammation and cancer, was not evaluated.

### 4.2 Polycystic kidney disease

Polycystic kidney disease (PKD) is a group of genetic disorders characterised by the progressive development of renal cysts. It can be autosomal dominant (ADPKD) or autosomal recessive (ARPKD), and the dominant form affects some 1 in 500–1000 people. A role for MYC in the pathogenesis of PKD was suggested by work using the spontaneous congenital polycystic kidney Cys1^cpk/cpk^ (*cpk*) mutant mouse that phenocopies human ARPKD. In this model, MYC overexpression was detected in polycystic kidneys, with only a minimal increase in proliferation, and also in collecting duct epithelial cells (Cowley et al., 1987; Harding et al., 1992). Notably, *in vivo* treatment with a *c-MYC* antisense oligomer (ASO) decreased *cpk* mouse kidney weight, improved their renal function and decreased the number of cysts, pointing to a therapeutic effect of MYC inhibition (Figure 3B) (Ricker et al., 2002).

Cystin, encoded by *Cys1*, is a lipid-microdomain associated protein found in the primary cilium of renal epithelia cells (Yoder et al., 2002) that binds to the *MYC* promoter and regulates its expression (Wu et al., 2013). While the loss of Cystin’s proper function increases MYC expression, transgenic complementation with Cystin-GFP expression rescues the phenotype with concomitant normalisation of MYC levels (Yang et al., 2021). Also, MYC is overexpressed in kidneys from ARPKD. More in detail, fibrocystin/poliductin protein localise to the nucleus, binds to *MYC* promoter P1 and activates its expression (Figure 3B) (Harafuji et al., 2023).

Others have reported further links between MYC and PKD. The SBM transgenic mouse model, with an SV40 promoter and beta-globin enhancer that drives MYC overexpression in renal epithelial cells, bears similarities with human ADPKD, which is mainly caused by mutations in the gene *PKD1*, encoding for polycystin-1 (PC1). These mice show significant upregulation of PC1 and develop PKD with 100% penetrance that leads to fatal renal failure. Examination of the kidneys revealed higher levels of MYC expression in the epithelial lining of cystic and hyperplastic tubules (Trudel et al., 1991).

*SBPkd1*_*TAG*_ mice overexpress *PKD1* mRNA leading to increased PC1 dosage in renal epithelial cells and exhibit a moderate rate of disease progression that leads to renal failure at five to six months. On the other hand, PC1 dosage-reduced *Pkd1*-cKO mice develop enlarged cystic kidneys that become very severe by P10 and leads to death due to renal failure. Puzzlingly, in both PC1 dosage-increased and -reduced mice, MYC expression (along with that of β-catenin) was found to be upregulated in renal cells with respect to wild-type mice. Moreover, MYC was found to be enriched in *PKD1* promoter regions in adult SBM mouse kidneys, while overexpression of MYC in HEK293 embryonic kidney cells increased the levels of PC1. These data suggest that *PKD1* expression is driven at least in part by MYC (Figure 3B) and unveils an inter-regulatory network involving MYC and PC1 that controls cystogenesis (Parrot et al., 2019).

While direct exogenous MYC inhibitors were not applied in these models, either genetic renal-specific ablation of MYC (Parrot et al., 2019), or treatment with inhibitors of BET bromodomain protein 4, an upstream regulator of MYC, reduced disease severity or delayed PKD progression (Zhou et al., 2015). Similarly, loss of MYC suppressed cystogenesis in a *Pkd1*-KO mouse model (Figure 3B) (Cai et al., 2018).

Combined, these data point to MYC as a causal cystogenic factor and a mediator of ADPKD. Its inhibition is therefore a potential therapy and further testing of inhibitors is highly warranted.

### 4.3 Multiple sclerosis

Multiple Sclerosis (MS) is an autoimmune disease of the central nervous system (CNS) characterised by the self-reactive T cell-induced demyelination. T cells recognise antigens on myelin basic protein, myelin oligodendrocyte glycoprotein and proteolipid protein, and immunization against these antigens induces the MS-like experimental autoimmune encephalomyelitis disease (EAE) in mice. More than 10 years ago, genome-wide association studies in MS patients identified single nucleotide polymorphisms in the *MYC* gene (International Multiple Sclerosis Genetics Consortium et al., 2011). In recent years, a series of papers have linked MYC’s transcriptional activity to T cell activation in MS.

First, MYC, together with NF-κB and mTOR, was found to be involved in the activation of memory Th and naïve T cells in EAE through the induction of PRMT5, an arginine methyltransferase that plays a crucial role in inflammatory T cell expansion and EAE disease (Webb et al., 2017; Webb et al., 2019). This constitutes another example of a positive interaction of MYC and mTOR, in contrast to UPR in osteoclastogenesis. Here, MYC’s role was demonstrated using the small molecule inhibitor 10058-F4.

Second, *MYC* transcriptional activation through phospho-STAT3 and RelA/NF-κB mediates T cell receptor-independent downstream signalling from activated CD28 that leads to inflammatory T cell responses in MS (Figure 3C) (Kunkl et al., 2019).

Finally, bioinformatic analyses of protein-protein interaction networks in MS found common genes and biological pathways for disease susceptibility, among which *MYC* was found to be a central gene in peripheral blood mononuclear cells from MS patients (Safari-Alighiarloo et al., 2020). A similar study confirmed the role of MYC, along with HNF4α and SP1, as a master regulator of CNS autoimmunity (Colombo et al., 2023). In this case, MYC was inhibited using OTX015, an inhibitor of BET domain proteins that indirectly decreases MYC levels. This inhibitor has been tested in clinical trials (in oncological indications), although it is not specific for MYC only. Further preclinical validation of the role of MYC and the potential of MYC inhibition in MS seems warranted.

### 4.4 Aging

Many of the biological processes implicated in or associated with aging have also been linked to MYC and its deregulation. These include the so-called hallmarks of aging (López-Otín et al., 2023): genomic instability, epigenetic alterations, stem cell exhaustion, energy production, protein translation, DNA damage, and inflammation. Transgenic mice have been used to explore the impact of systemic MYC level reduction, but so far there are contrasting results. Initially, MYC haploinsufficiency studies showed that *MYC* ^+/−^ mice had significantly extended lifespans with amelioration of aging phenotypes across a variety of pathophysiological processes when compared to wild-type littermates. These included healthier lipid and cholesterol metabolism, less fibrosis and cancer progression, higher metabolic rate and less immunosenescence. The exact mechanism(s) behind this have not yet been established, although they are expected to be multifactorial, through decreased expression of direct MYC targets, or indirectly through other transcription factors and/or miRNAs regulated by MYC. For instance, ribosomal *RPL* and *RPS* genes were found to be reduced in MYC haploinsufficient tissues with the concomitant reduction of *in vivo* translation, which is clearly associated with longer lifespan (Figure 3D) (Hofmann et al., 2015). Additionally, these mice showed decreased systemic levels of IGF1 through MYC-miR122 regulation. Reduced IGF1 has been linked to the development of age-related diseases such as osteoporosis, but female MYC haploinsufficient mice had a decreased incidence of osteoporosis, consistent with the finding that, in bone, IGF1 levels were unaltered (Petrashen et al., 2023).

The current understanding of aging considers it as a multifactorial process in which different signalling pathways converge on autophagy genes to regulate lifespan. WIPI1, and its *C. elegans* ortholog ATG-18, has been identified as one of the critical autophagic factors involved in extending lifespan (Tóth et al., 2008). In line with this and with MYC’s supposed role in aging, it was found that the ABL-MYC axis represses WIPI1 gene expression. Interfering with this axis promotes autophagy and extends *C. elegans* lifespan (Sporbeck et al., 2023).

On the other hand and in contrast with the results above, transgenic mice engineered with near-complete elimination of MYC at weaning, named *MycKO* mice, aged prematurely yet lived longer with decreased cancer incidence. The phenotypic alterations were, as expected, copious and broad: bone marrow hypoplasia, peripheral cytopenia, alopecia, achromotrichia, glucose intolerance and mitochondrial dysfunction. Additionally, colonic epithelial flattening and villous atrophy were found, although there was no effect on body weight (Prochownik and Wang, 2023; Wang et al., 2023).

Hence, according to these latter results, aging appears to be associated with higher cancer incidence only in the presence of MYC (Figure 3E). It remains to be seen what effect chronic administration of MYC inhibitors could have on indicators of aging. Whether such chronic treatment could also be applied as a cancer prevention strategy (and not only to cancer treatment) is not clear beyond preclinical models.

### 4.5 Cardiac metabolism after pathological stress

Cellular oxygen concentrations are tightly regulated in eukaryotic organisms to maintain proper mitochondrial function and energy production. Mammalian cells adapt to oxygen deprivation by inducing protective mechanisms. For instance, a substantial decrease in protein biosynthesis is among the effects of hypoxic stress on cardiomyocytes, where transcription factor IIIB (TFIIIB) and TFIIIC-dependent RNA polymerase III (pol III) play a key role (Kraggerud et al., 1995; Schramm and Hernandez, 2002). *In vitro* experiments with neonatal rat myocytes at 1% O_2_ revealed that HIF-1α induces the dissociation of MYC from TFIIIB, contributing to the decrease in pol III transcription (Ernens, 2006). Other pathological stressors such as haemodynamic load and ischemia divert metabolic pathways away from fatty acid oxidation (FAO) towards glucose metabolism (Stanley et al., 2005). In the adult heart, this metabolic rewiring in the myocardium is mediated by MYC, whose increased levels downregulate genes involved in FAO, while concomitantly upregulating genes mediating glucose oxidation, such as *ENO1*, *PFKM*, *LDHA* and SLC16A1 (Figure 3F). This was associated also with an increase in the number of functional mitochondria and represents MYC-dependent metabolic adaptation towards a better response to ischemic insults (Ahuja et al., 2010).

Cardiac progenitor cells (CPCs), however, become quiescent after ischemic hypoxia, limiting their self-renewal and vasculogenic properties, with the aim of preserving stem cell homeostasis (Guitart et al., 2010). Being a master regulator of the cell cycle and quiescence, it is no surprise that MYC, after *in vitro* hypoxia (0.5% O_2_), is downregulated in mouse CPCs isolated from the myocardium, with a concomitant increase in the levels of the CDK inhibitor p21, a MYC target (Bellio et al., 2017).

Neonatal cardiac proliferative potential is lost after a week, coinciding with downregulation of multiple genes involved in cell cycle, including *MYC* (Walsh et al., 2010; Quaife-Ryan et al., 2017). Ectopic cardiac MYC-dependent transcription and cell cycle progression in the adult heart *in vivo* depends on the levels of P-TEFb, a protein complex consisting of CDK9 and Cyclin T1. In order to effectively drive cell division in the heart, MYC expression must be accompanied by higher levels of P-TEFb (Bywater et al., 2020). In line with this, transient expression of both MYC and Cyclin T1 by a single intramyocardial dose of a modified RNA coding for both genes was shown to be a potential regenerative therapeutic in the heart after myocardial infarction, inducing cell cycle and division of cardiomyocytes (Figure 3G) (Boikova et al., 2023).

Restoration of reperfusion is the most effective treatment for myocardial infarction. Paradoxically though, reperfusion leads to myocardial ischemia/reperfusion (MI/R) injury, which induces cardiomyocyte apoptosis through increased oxidative stress (Wang et al., 2017). Using an MI/R mouse model, MYC was found to be downregulated, with consequent oxidative stress and cardiomyocyte apoptosis (Wen et al., 2022). Notably, therefore, recovery after ischaemia using these regenerative or protective strategies represents one of the few conditions in which therapy would require MYC expression or activation.

Hypertension is one of the most common pathologies of the vascular system. It leads to overload, increasing the risk of myocardial infarction, among others. The myocardium of spontaneously hypertensive rats (SHRs) overexpresses MYC and its downstream target CYP2E1, whose overexpression leads to oxidative stress and other pathological processes. Long-term treatment with quercetin, a flavonoid with potential cardiovascular beneficial effects, resulted in a significant reduction of blood pressure with concomitant downregulation of MYC and CYP2E1, significantly improving the prooxidant-antioxidant profile (Maksymchuk et al., 2023). Whether downregulation of MYC alone would reduce blood pressure, CYP2E1 expression, and curb the oxidative stress, still remains to be seen.

### 4.6 Restenosis

The arterial wall response to pathophysiological stimuli, including atherosclerosis and angioplasty procedures, involves the proliferation of smooth muscle cells (SMC). Indeed, 25%–50% of patients undergoing angioplasty will develop recurrent stenosis, which is essentially a narrowing of the blood vessels, also called restenosis, that consists of the proliferation of medial SMC and their migration to the subintima. Because of this, considerable attention has been paid to the inhibition of SMC proliferation as a way of preventing restenosis. Initial studies involving antisense oligonucleotides (ASOs) targeted SMC *PCNA in vitro* with significant inhibition of proliferation (Speir and Epstein, 1992). Much later studies focused on the use of a phosphorodiamidate morpholino oligomer (PMO) antisense to the c-MYC translation initiation site, called AVI-4126 (Resten-NG^®^) (Figure 3H). It was successfully tested in a rabbit balloon injury model (Kipshidze et al., 2002) and porcine restenosis model (Kipshidze et al., 2003; Kipshidze et al., 2002) with promising results: significant reduction of the neointimal area with concomitant MYC inhibition. Although this was further validated in a Phase II trial with positive results (Philipp, 2012), the drug was not developed beyond this point.

### 4.7 Bone developmental disorders

Septic nonunion (SN) is a bone disorder caused by the failure of fracture healing. It is often caused by local inflammation. Expression of the lncRNA *RUNX2-AS1* was detected in SN biopsies, along with proinflammatory cytokines. *RUNX2-AS1* negatively regulates *RUNX2* expression and its downstream targets, which play an important role in bone differentiation and development. It was found that MYC associates with MAX, p300 and NCOA2 to induce RUNX2-AS1 expression, abrogating the expression of RUNX2 target genes, while LPS-induced inflammation induced the expression of NCOA2 and showed a dose-dependent increased association with MYC-MAX-p300. These results link the inflammatory microenvironment with the downregulation of RUNX2 and its target genes, which impairs bone differentiation and leads to nonunion (Li and Qian, 2022).

Ankylosing spondylitis (AS) is a heritable chronic inflammatory disease that affects the spine and pelvis, ultimately leading to joint ankylosis due to ectopic ossification and disability. Inflammation is an early characteristic of AS and inflammatory cytokines could promote ossification by modulating the osteoblasts (Li et al., 2020). MYC was found to be upregulated in AS ligament samples and in fibroblasts in an *in vitro* osteogenic model. In this model, two osteogenic genes were found to be dependent on MYC: alkaline phosphatase (*ALP*) and bone morphogenetic protein 2 (*BMP2*). Additionally, the inflammatory cytokines IL-23 and IFN-γ upregulate both *MYC* and *ALP in vitro*. In AS ligament samples, a higher proportion of IL-23 positive and IFN-γ positive cells were found with respect to osteoarthritis samples (Figure 3I) (Jin et al., 2023). Osteoporosis has also been linked to MYC. Based on bioinformatic analysis, a series of experiments showed that the MYC/ERRα axis regulates mitochondrial respiration in osteoclastogenesis, and their targeting protected mice of oestrogen deficiency-mediated bone loss after ovariectomy, pointing to MYC as a potential therapeutic target for osteoporosis (Bae et al., 2017).

### 4.8 Potential role of MYC in other diseases and conditions

Various studies link MYC to a range of other disorders, mainly through experiments to determine its expression in model systems or patients. In particular—and not surprisingly—there are strong suggestions of a role in endometriosis (Nothnick et al., 2023), mitochondrial diseases (Purhonen et al., 2023), immune-related, neurodegenerative and other metabolism-related diseases. These are summarised in Table 1 with some of the data hinting at a role for MYC. In general, more work is required to prove a clear link and determine whether MYC is playing a role in disease causation, or even whether modulation of its expression could be preventative.

**TABLE 1:** Additional diseases and conditions in which MYC has been implicated. In these cases, the evidence is more preliminary than for those described in the main text. We have indicated studies showing any links between MYC and the disease, and in particular, any data regarding MYC modulation, either by inhibition or overexpression.

Disease or condition	Studies linking it to MYC	MYC modulation experiments
Neurodegeneration 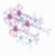	• Phosphorylated c-MYC, c-MYC and N-MYC found in patient samples from various neurodegenerative diseases (Ferrer and Blanco, 2000; Ferrer et al., 2001)	• MYC inhibition by mithramycin (a non-selective inhibitor) was neuroprotective (Sleiman et al., 2011)
		• Downregulation of dMyc is neuroprotective in tauopathies in *Drosophila* (Chanu and Sarkar, 2017)
		•Expression of Myc induces ND in transgenic models (Lee et al., 2009)
Neuropathic pain 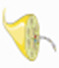	• MYC expression in mouse models (Van et al., 2010; Jiang et al., 2022)	• MYC overexpression induces pain hypersensitivity while knockdown *in vivo* with siRNA alleviates neuropathic pain (Jiang et al., 2022)
Acute liver failure 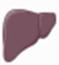	• MYC-dependent transcriptional program orchestrates cell activation during ALF (Kolodziejczyk et al., 2020)	• MYCi in mouse models using small molecule Kj-Pyr-9 attentuates ALF (Kobdziejczyk et al., 2020)
Diabetic nephropathy 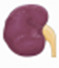	• Increased MYC expression in endothelial cells in response to glucose, and in DN patients and rats (Hou et al., 2022)	• MYC overexpression or siRNA knockdown impacts endothethial cell inflammation (Hou et al., 2022)
	• N-MYC stabilisation in models (Choi et al., 2023)	
	• MYC is one of the top Differentially Expressed Genes in glomerular samples of patients (Hojjati et al., 2023)	
Fanconi anemia 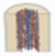	• High levels of MYC mRNA in primary stem cells from patients and bone marrow, and nuclear MYC IHC in liver sections (Rodriguez et al., 2021)	• JQ1 inhibitor reduces MYC expression, and decreases clonogenic potential and genotoxic stress in stem cells from FA-mice (Rodriguez et al., 2021)
Diabetes 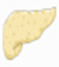	• MYC increased in mouse and rat diabetes models (Jonas et al., 1999; Jonas et al., 2001; Laybutt et al., 2003)	• MYC overexpression in beta-cells triggers diabetes in mouse models (Laybutt et al., 2002)
	• Upregulation of c-MYC/N-MYC networks in proteome and transcriptome analysis of non-obese diabetic mice (Gerling et al., 2006; Wu et al., 2013)	• MYC induction upon BCG vaccination improves glucose metabolism (KOhtreiber et al., 2020)
	• MYC gene network and protein increased in diabetic patients (Kaizer et al., 2007; Lee et al., 2008)	
	• c-Myc directly induces both impaired insulin secretion (Kaneto et al., 2002), potentially through PKC (Kaneto et al., 2002) and loss of 0-cell mass, independently of hyperglycemia (Cheung et al., 2010)	
Kefold scar 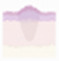	• MYC gene expressed increased in microarray analysis of patient skin (Chen et al., 2004)	• c-MYC overpexpression promotes the proliferation of keloid fibroblasts (Feng et al., 2023; Piao and Feng, 2023)
	• MYC protein expression increased in patient samples (Hu et al., 2002; Zhang et al., 2023)	
Developmental diseases 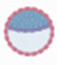	• Cornelia de Lange syndrome and Roberts syndrome are linked to misregulation of MYC (Horsfield et al., 2012)	Not found
	• Feingold disease is caused by MYCN haploinsufficiency (van Bokhoven et al., 2005; Cognet et al., 2011)	
	• Achondroplasia linked to MYC downregulation (Zhou et al., 2011)	
Irritable Bowel Disease 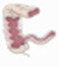	• MYC amplification in IBD-associated carcinoma patients (Hartman et al., 2018)	Not found
	• Some MYC IHC in IBD-associated low-grade dysplasias (Liang et al., 2023)	
Immune related diseases		
Systemic lupus erythematosus 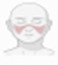	Not found	MYCi by JQ1 abolishes the pathogenic response induced by functional Breg cells (Wang et al., 2022)
Uveitis 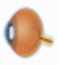	• MYC increased in experimental autoimmune uveitis (Chen et al., 2022)	• MYC knockdown reduces miR-181a-5p, involved in the pathogenic Th17 immune response (Chen et al., 2022)
Arthritis 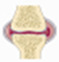	• MYC expression increased in synovial tissue microarray analysis of OA patients (Zhang et al., 2023)	• Simultaneous inhibition of both c-Myc (with 10,074-G5) and HIF-la is efficacious for anti-inflammation *in vitro* and *in vivo* in RA model (Lee et al., 2020)
	• MYC gene expression is increased in RA patients (Harshan et al., 2022; Fan et al., 2023)	
Pancreatitis 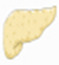	Not found	• MYCi by 10,058 reduces markers of acute pancreatitis in mouse models (Xu et al., 2020)
Asthma 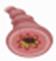	• MYC gene and network upregulated in patients (Troy et al., 2016; Vargas et al., 2016; Salameh et al., 2022)	• iPSC-w/o-c-Myc transplantation had therapeutic effects in allergic airway hyperresponsiveness (Wang et al., 2013)
	• Higher MYC expression in inflammatory cells in allergic asthma mouse model (Shen et al., 2019)	
	• MYC upregulation involvement in pathogenesis of ILC2 in asthma (Ye et al., 2020)	
	• Wnt/p-catenin regulate asthma airway remodeling and upregulate c-MYC (Jia et al., 2019)	
Coeliac disease 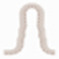	• MYC expression increased mouse models (Vaira et al., 2020) and patient samples (Ciclitira et al., 1987)	Not found
infections 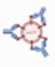	• MYC as a hub gene in tuberculosis (Xiao et al., 2023)	• MYC activation-deficient adenovirus impairs glutamine catabolism needed for viral replication and infection of primary cells (Thai et al., 2015)
	• Wnt6 increases MYC expression in granulomatous lesions of *Mycobacterium* in the lung (Schaale et al., 2013)	• MYC expression rescues *Chlamydia* persistence in cell lines (Vollmuth et al., 2022)
	• SARS-CoV-2 Orf7b protein upregulates MYC which mediates lung apoptosis and ferroptosis (Deshpande et al., 2024)	

## 5 Current state of MYC inhibition in the clinical setting

**FIGURE 4:**
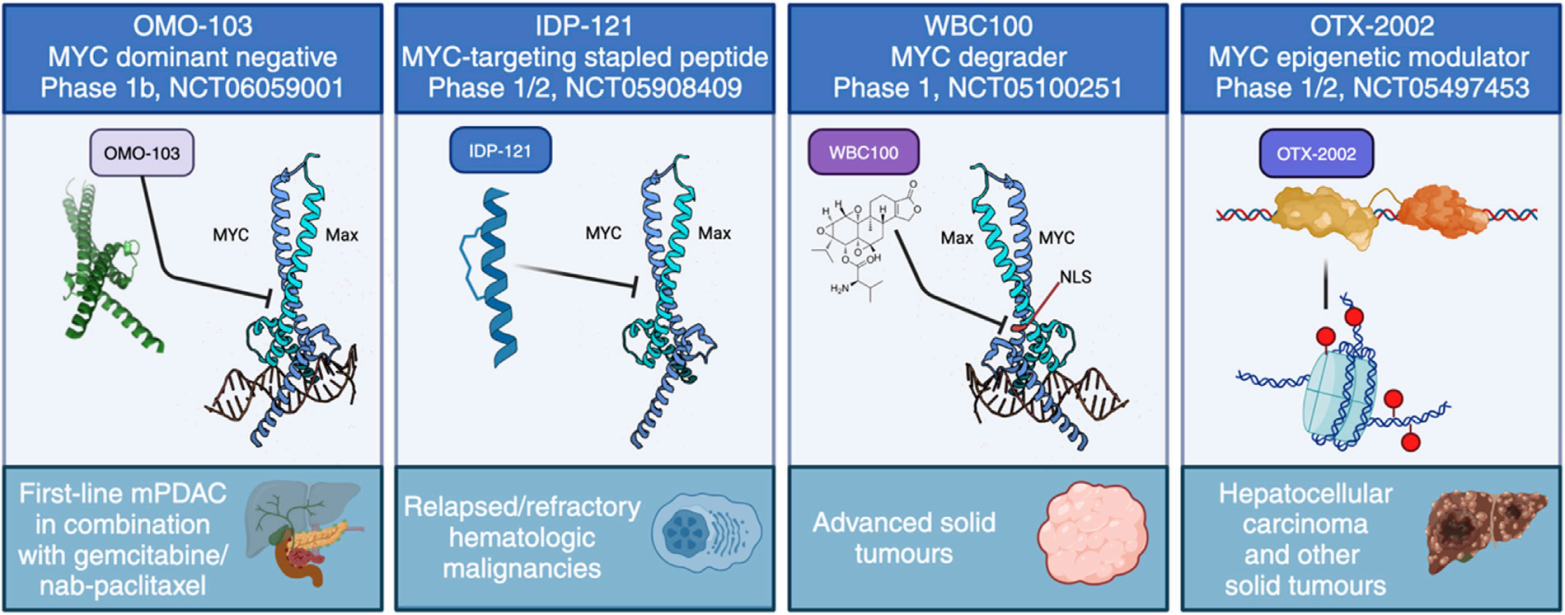
The current approaches to directly target MYC in clinical trials. These include four distinct strategies: MYC dominant negative OMO-103, anti-MYC stapled peptide IDP-121, MYC degrader WBC100 and MYC epigenetic modulator OTX-2002.

There are a huge number of reviews describing the search for MYC inhibitors and their application to cancer treatment. Here we will only briefly describe some targeting strategies, focusing on those reaching clinical testing, summarised in Figure 4, and refer the reader to a number of other much more in-depth reviews regarding MYC inhibitors, both from our group (Whitfield et al., 2017; Massó-Vallés and Soucek, 2020; Whitfield and Soucek, 2021; Martínez-Martín et al., 2023) and others (Ross et al., 2021; Karadkhelkar et al., 2023; Weber and Hartl, 2023).

Strategies employed fall into two main approaches: direct and indirect inhibitors. The latter include a much more expansive set of possibilities, since their target can be anything that interacts with MYC or controls its activity, expression, or localisation. These could also include synthetic lethal targets: here, any protein or signalling pathway that is essential for the survival of MYC-driven tumour cells can be targeted and many such targets are in clinical development. Direct inhibitors, on the other hand, impinge on MYC itself to control the expression or stability of the RNA or protein, or its interaction with DNA or dimeric partners.

Perhaps surprisingly, the earliest MYC inhibitors to be tested in clinical trials were applied to a non-oncological indication (Kipshidze et al., 2002). Antisense oligonucleotides (ASOs) were used for the treatment of heart restenosis (NCT00244647, NCT00248066) (Kipshidze et al., 2007). These showed some positive effects in this coronary disease and were later tested against neoplasms, showing significant tissue accumulation in solid tumours (Devi et al., 2005), but to our knowledge no further development occurred.

Other more recent trials have also been discontinued, including: Quarfloxin (CX-3543) and APTO-253, G-quadruplex stabilisers thought to work by preventing *MYC* transcription (NCT00780663, NCT02267863); INX-3280, another ASO (Kutryk et al., 2002); and DCR-MYC, an siRNA to prevent *MYC* translation (NCT02110563, NCT02314052). These were all tested as cancer therapies (reviewed in Whitfield et al., 2017), but none was further pursued.

The first successful Phase I trial has recently concluded using OMO-103, a MYC dominant negative mutant based on the Omomyc mini-protein, delivered intravenously once per week. In line with its extensive preclinical validations, OMO-103 showed a good safety profile and some first hints at efficacy in all-comers solid tumours (Garralda et al., 2024). In addition, biomarkers were identified indicating MYC inhibition. A Phase Ib trial recently started in September 2023 in metastatic pancreatic cancer (NCT06059001).

To our knowledge, there are currently three other ongoing trials with a direct MYC inhibitor: one uses a MYC degrader called WBC100, in MYC-positive advanced solid tumours (NCT05100251), another is an epigenetic controller, OTX-2002, that downregulates MYC and is being tested in hepatocellular carcinoma (NCT05497453), while the third is with IDP-121, a stapled peptide MYC inhibitor being evaluated in patients with relapsed/refractory hematologic malignancies (NCT05908409). In addition, MYC-related indirect approaches have reached clinical trials. For instance, MYC-induced protein translation depends on GSPT1, and a molecular glue degrader of GSPT1 (MRT-2359) is currently being trialled for MYC-driven and other selected solid tumours (NCT05546268). Still, the focus remains firmly on testing in cancer.

As discussed already, any approved inhibitor could potentially be applied to other non-neoplastic conditions.

## 6 Possibilities to activate or express MYC

As explained in the previous section, the majority of examples of MYC involvement in diseases point to its inhibition as a therapeutic approach. However, MYC activation could be an option to favour regeneration in the heart after myocardial infarction or hypoxia. Further to such repair and regeneration approaches, a recent study highlighted the use of MYC activation by transgenic overexpression to stimulate *ex vivo* platelet production from induced pluripotent stem cells (Kayama and Eto, 2024). This could eventually provide improved transfusion systems. Additionally, an unexpected indirect approach could benefit Type 1 Diabetes (T1D) patients, in which the administration of BCG vaccine resulted in long-lasting blood sugar control with proper glucose metabolism (Li et al., 2018). A recent study found a gradual *MYC* mRNA upregulation in monocytes and CD4 T cells from T1D patients. This led to increased transcription of MYC-dependent glucose and glutamine metabolism genes (Kühtreiber et al., 2020).

## 7 Perspective

While MYC has long been considered an undruggable target, new therapeutic options against it are becoming clinically viable, as demonstrated by the completion of the first successful clinical trial of a direct inhibitor, OMO-103. Most of the trials and recent focus remains in the field of cancer treatment, and indeed the ongoing trials of direct MYC inhibitors are against PDAC, hepatocellular carcinoma, relapsed/refractory hematologic malignancies, and MYC-positive advanced solid tumours. As mentioned in this review, though, MYC’s pleiotropic roles in multiple physiological processes suggest that its modulation could be applied to many other diseases. To date, there is preliminary data pointing to a role in a variety of diseases of different origins and clinical presentations such as neurodegeneration, diseases of the bone, digestive system and related organs, keloid scars, developmental and immune-related diseases (such as asthma, coeliac disease, and others), as well as the aging process. Further pre-clinical testing and even clinical trials seem merited in these cases.

In general, excess or over-active MYC is detrimental, so under physiological circumstances its levels are precisely controlled to keep the multiple downstream processes in check. In most diseases described so far, and as seen in cancer, where deregulation of MYC is frequent, such excessive MYC activity drives various processes that then lead to pathologies due to the unfettered proliferation, changes in differentiation and altered metabolism, among others. There is huge potential, therefore, for using MYC inhibitors that are currently being developed in the cancer field.

Diseases in which MYC activation may instead be desired include those where stimulation of cell proliferation and tissue regeneration is needed, such as after ischaemic damage in the heart, diabetes, and neuronal repair. It has been speculated that in neurodegeneration, MYC activation may be part of a failed neuroprotective response. Thus, extra MYC could help repair and regenerate neurons after cell death or damage. Of note, activation of MYC for such diseases will likely be required locally, in the affected tissues rather than systemic, to avoid the foreseeable massive and deleterious effects that body-wide activated MYC could have.

In summary, if we have learnt something from 40 years of literature about MYC, it is that we still have a lot to discover. Luckily, pharmacological tools for its modulation seem finally viable and hold promise for a better understanding of MYC biology, while also providing the basis for new therapeutics applicable to multiple indications in oncology and beyond.
